# Modulation of Vascular ACE by Oxidative Stress in Young Syrian Cardiomyopathic Hamsters: Therapeutic Implications

**DOI:** 10.3390/jcm5070064

**Published:** 2016-07-13

**Authors:** Nildris Cruz, Jorge D. Miranda, Maria J. Crespo

**Affiliations:** 1 Department of Physiology, University of Puerto Rico-School of Medicine, San Juan 00936, Puerto Rico; nildris.cruz@upr.edu (N.C.); jorge.miranda3@upr.edu (J.D.M.); 2 Department of Anesthesiology, University of Puerto Rico-School of Medicine, San Juan 00936, Puerto Rico

**Keywords:** ACE, NOS, endothelial dysfunction, Syrian cardiomyopathic hamsters, Heart failure

## Abstract

Increased vascular angiotensin-converting enzyme (ACE) activity and oxidative stress are present in young Syrian cardiomyopathic hamsters (SCH) before the clinical manifestation of heart failure (HF). The developmental time-course of these alterations and their potential interactions, however, are still unknown. We evaluated mRNA and protein levels of ACE, endothelial nitric oxide synthase (eNOS), and inducible nitric oxide synthase (iNOS) in the vasculature of SCH from one to four months of age. Total RNA and proteins were quantified with real-time reverse transcriptase-polymerase chain reaction (RT-PCR) and Western blot, respectively. The role of nitric oxide (NO) on vascular ACE activity was also assessed. ACE mRNA and protein levels were up-regulated in SCH at two months of age compared with controls (CT) (*p* < 0.05). At this two-month stage, eNOS protein levels were lower in SCH (87%) than in CT (100%) (*p* < 0.05), although iNOS protein levels increased significantly (482%) compared to CT (100%; *p* < 0.05). In addition, ACE mRNA expression and activity were modulated by NO at two months of age. Thus, the combination of low eNOS and high iNOS protein levels may underlie vascular renin-angiotensin system (RAS) over-activation. Altogether, these factors may contribute to the development of endothelial dysfunction and vascular hyper-reactivity in the early stages of heart failure, and eventually trigger cardiac deterioration in this animal model of HF.

## 1. Introduction

Heart failure (HF) is a complex syndrome in which the capacity of the heart to pump blood is decreased, leading to a mismatch between cardiac output and the metabolic demands of tissues. Although the precise mechanisms involved in the etiology of HF remain unclear, over-activation of the renin-angiotensin system (RAS) and the sympathetic nervous system is a known hallmark of this condition [1]. Endothelial dysfunction and oxidative stress are also commonly found in HF [2]. The temporal relationship between the appearance of these alterations and the development and progression of HF, however, remains unknown.

Endothelial dysfunction, which is characterized by an impaired ability of acetylcholine to relax blood vessels, may result from either a decrease in nitric oxide (NO) bioavailability or its production, or both [3]. Increased angiotensin II levels may reduce NO bioavailability by activating NADPH oxidase, thereby favoring the production of reactive oxygen species (ROS), which are NO scavengers [4]. Alterations in NO synthase (NOS) isoforms may also contribute to decrease NO levels. Uncoupling of endothelial nitric oxide synthase (eNOS) or up-regulation of inducible nitric oxide synthase (iNOS) reduces NO by stimulating ROS formation and promoting oxidative stress, thereby increasing the risk of cardiac damage [5]. That angiotensin-converting enzyme (ACE) inhibitors enhance eNOS expression and NO bioavailability suggests an interaction between RAS and oxidative stress [6]. Indeed, cross-talk regulation between eNOS expression/activity and tissue ACE expression/activity has been described for hypertensive rats [7]. This regulation appears to be bidirectional and counter-regulatory [7]. A direct association between NO availability and ACE activation, however, has not been established in HF, although decreased NO levels are common in patients and animal models with this condition, which show increased vascular ACE activity [8,9,10]. Thus, reductions in NO at the vascular level may play a central role in the activation of ACE.

Previous studies from our laboratory demonstrated that in the two-month-old Syrian cardiomyopathic hamster (SCH), an animal model of dilated cardiomyopathy and HF, vascular abnormalities precede cardiac dysfunction [9,10,11,12,13]. These abnormalities include increased vascular ACE activity [10], ROS-mediated endothelial dysfunction in the coronary circulation [13], and impaired acetylcholine-induced relaxation in aortic rings [9]. The impaired relaxation was due to endothelial dysfunction, not to alterations in the vascular smooth muscle, because concentration-response curves with the NO donor sodium nitroprusside were similar in SCH and CT [9]. RAS and oxidative stress are enhanced in the vasculature of young SCH (two months old) before the onset of HF, suggesting that the vasculature plays a crucial role in the development and progression of this condition. Mollnau and colleagues [14] demonstrated that the expression of eNOS, the main regulator of NO production, is reduced in the vasculature of old (10-month-old) SCH. Chronic treatment of these animals with the ACE inhibitor captopril increases this expression [14]. The latter finding suggests an interaction between vascular ACE and eNOS in old animals. Whether this interaction is also present in young animals is still unknown. That the blockade of RAS with losartan reduces oxidative stress in the vasculature of two-month-old SCH [11] suggests that the interaction between ACE and NO-producing systems is present at the early stages of development, and precedes cardiac deterioration. Clarifying the timeline for RAS activation, the status of NOS isoforms, and the relationship between these two systems in the vasculature of young SCH may provide insight into the etiology of cardiac deterioration, and contribute to the development of new therapeutic strategies for HF treatment.

The present study investigates ACE, eNOS, and iNOS mRNA expression and protein levels in the vasculature of young (one to four months) SCH, prior to the appearance of HF. We found increased ACE activity, mRNA expression, and protein levels concomitant with reduced eNOS protein and increased iNOS protein in the vasculature of two-month-old SCH. Together, these factors may underlie endothelial dysfunction and vascular hyper-reactivity, both of which precede cardiac deterioration in young SCH.

## 2. Experimental Section

### 2.1. Animal Model

Male Syrian cardiomyopathic hamsters (SCH, Bio-TO2 strain) and golden control hamsters (CT, F1-B strain) were purchased from Bio Breeders, Inc. (Boston, MA, USA) at one, two, three and four months of age. The hamsters were housed individually in a temperature-controlled room under a regular light-dark cycle (12L: 12D, lights off at 5:00 p.m.). Water and food (Harlan Rodent Diet, 18% protein) were provided ad libitum. All experiments were approved by the Institutional Animal Care and Use Committee (Protocol #2590105), and adhered to the Guide and Care for the Use of Laboratory Animals published by the National Institutes of Health (Bethesda, MD, USA) and the American Veterinary Medical Association.

### 2.2. RNA Extraction and Real-Time RT-PCR

The day of the experiments, animals were anesthetized with an intraperitoneal (IP) injection of sodium pentobarbital (50 mg/kg). When complete anesthesia was achieved, animals were transcardially perfused with ice-cold 0.01 M phosphate-buffered saline (PBS), pH 7.4 (Sigma-Aldrich, St. Louis, MO, USA). After perfusion, the aorta was removed and placed in RNAlater (Ambion RNA stabilization solution; Ambion Inc, Austin, TX, USA), and stored at −80 °C as described by Santiago and colleagues [15]. Total RNA was extracted using Trizol (Sigma-Aldrich, St. Loius, MO, USA) and the samples then were treated with DNAse I to avoid genomic contamination. The integrity of the total RNA was verified by electrophoresis in a 1% agarose-formaldehyde gel, and total RNA quantification was performed by absorbance at 260 nm. cDNA synthesis was performed with 1 µg of RNA, using the iScript cDNA Synthesis Kit (Bio-RAD Laboratories, Hercules, CA, USA). Negative controls (mock samples) were prepared without Reverse Transcriptase added to cDNA synthesis reaction to discard the presence of genomic contamination.

Real-time RT-PCR studies were conducted using a modified protocol described previously [16]. Gene analyses to select specific primer sequences were performed with Beacon Designers 3 (Primer Biosoft International, Palo Alto, CA, USA). The specific primers for ACE, eNOS, iNOS and elongation factor 1-α (EF1-α genes were designed and manufactured by IDT^®^ Integrated DNA Technologies Inc. (Coralville, IA, USA). The primer sequences and annealing temperatures for ACE, iNOS, eNOS and EF-1α are described in Table 1. Amplifications reactions included sense/antisense primer mix (10 µM), cDNA (100 ng) and the iQ™ SYBR^®^ Green Supermix kit (Bio-Rad, Hercules, CA, USA), which contains a 2× reaction buffer with Tris-HCl (40 mM, pH 8.4), KCl (100 mM), dNTPs mixture (0.4 mM each), MgCl2 (6 mM), iTaq DNA polymerase (50 u/mL), and SYBR Green I (20 nM). An iCycler (Bio-Rad, Hercules, CA, USA) was used to perform the Real-time RT-PCR reaction in the following sequence: a hot-start for 3 min at 95 °C; 40 cycles of denaturing for 30 s at 95 °C; exposure for 1 min to an annealing temperature (Table 1); an extension step for 1 min at 72 °C; and a final extension step for 5 min at 72 °C. Amplification of EF1-α was used as a housekeeping gene to monitor the specificity of any change in gene expression. The presence of a single PCR product was verified, using melt curve and gel analyses. The identity of the PCR fragment was confirmed by sequencing the purified fragment.

### 2.3. Protein Extraction and Western Blot

Each animal was anesthetized with an injection of sodium pentobarbital (IP, 50 mg/kg). The aorta then was carefully removed and stored at −80 °C. The tissue was processed in a 10 mM Tris- 1 mM EDTA buffer (pH 7.4) containing proteases and phosphate inhibitors: 5 mM NaF, 2 µg/mL antipain, 10 µg/mL aprotinin, 5 mM benzamidine, 1 mM dl-Dithiothreiol (DTT), 10 µg/mL leupeptin, 1 mM Na_3_VO_4_, 1 mM phenylmethanesulfonyl fluoride (PMSF), and 10 µg/mL trypsin inhibitors. The tissue samples were mechanically homogenized with scissors and sonicated (Vibracell vcx130, Milltown, CT, USA) at 45% amplitude for 90 s (3 × 30 s), with a 30 s resting period between each sonication cycle. The homogenate was centrifuged for 20 min at 14,000 rpm at 4 °C, and the supernatant fraction was collected and quantified, using the Bradford assay.

Western blot studies were performed with a modified protocol described previously [17]. Protein samples were separated by electrophoresis in a 6% SDS-PAGE gel. Proteins (7.5–10 µg) that are within the linear range of detection were then transferred to nitrocellulose membranes. Then, the appropriate transfer of proteins to nitrocellulose membranes was verified by staining in Ponceaus S (0.1% in glacial acetic acid) for 10 min and then washed in PBS. The membranes were blocked with 5% Blotto (5% powdered non-fat milk, 0.1% Tween-10%; Bio-Rad, Hercules, CA, USA) in Tris-NaCl (10 mM Tris-HCl and 100 mM NaCl; Sigma-Aldrich, St. Louis, MO, USA) for 1 h. Mouse monoclonal antibodies for eNOS (1:2000; BD Biosciences, San Jose, CA, USA), iNOS (1:1000; BD Biosciences, San Jose, CA, USA) and goat polyclonal antibody for ACE (1:2000; Santa Cruz Biotechnology, Santa Cruz, CA, USA) were added to the membranes after dilution in Blotto, and incubated overnight at 4 °C. The membranes were washed and then incubated with the secondary anti-mouse or anti-goat antibody (1:4000; Santa Cruz Biotechnology, Santa Cruz, CA, USA) coupled to Horseradish Peroxidase (HRP) for 1 h at room temperature. Before exposure and development, the membranes were washed and then incubated with the Super Signal West Femto Maximum Sensitivity Substrate (Thermoscientific, Waltham, MA, USA) to enhance the HRP signal derived from the secondary antibody according to the manufacturer’s instructions. The development and analysis of the membranes were performed with the Versadoc™ Imaging System and Quantity One Software (Bio-Rad Laboratories, Hercules, CA, USA). The levels of ACE, eNOS, and iNOS were standardized to the amount of β-actin housekeeping gene detected (1:4000: Sigma-Aldrich, St. Louis, MO, USA).

### 2.4. Evaluation of Vascular ACE Activity

To determine whether vascular ACE over-activation in SCH responds to changes in NO levels, we tested the effect of l-NAME, an inhibitor of NOS, and sodium nitroprusside (SNP), an NO releaser on ACE activity. Two-month-old SCH and CT hamsters were anesthetized with sodium pentobarbital (IP, 50 mg/kg). The aortas were removed and carefully cleaned to avoid damaging the smooth muscle and endothelium. Isolated aortas, approximately 1 cm of length, were placed in organ chambers and aerated (95% oxygen and 5% carbon dioxide mixture) at 37 °C in a Kreb’s bicarbonate solution (118 mM of NaCl, 2.5 mM of CaCl2, 5 mM of KCl, 1.1 mM of MgSO_4_, 25 mM of NaHCO_3_, 1.2 mM of KH_2_PO_4_, and 10 mM of glucose; pH = 7.4). The chambers also contained either 1 mM l-NAME or 1µM SNP. The aortas were incubated in the chambers for 6 h. After the incubation period, vascular ACE activity and mRNA were evaluated as described previously [18]. ACE activity was determined in aorta homogenates of untreated and treated SCH and CT hamsters. Homogenates were prepared by adding aortic tissue to 2 mL of ice-cold potassium phosphate buffer (50 mM, pH 7.5). A 100 µL aliquot of the homogenate was then added to 100 µL of Hip-His-Leu (Sigma-Aldrich, St. Louis, MO, USA) and a final volume of 250 µL was achieved by adding 50 µL of water. The mixture was incubated for 10 min at 37 °C. The reaction was then stopped with 1.45 mL of 280 mM NaOH. After this stage, 1% phthalaldehyde was added, the mixture was incubated for 10 min at room temperature, and 200 µL of HCI (3N) was added to the mixture. After a final 30 min incubation period, the medium was excited at 364 nm, and the fluorescence was determined at 486 nm, using a SpectraMax M3 Microplate Reader (Molecular Devices, Silicon Valley, CA, USA). To quantify ACE activity, a calibration curve was performed, using variable His-Leu concentrations from 0 to 5.0 mM.

### 2.5. Statistical Analysis

Results are presented as the Mean ± SEM. Student’s t-test was used to compare two groups, whereas the analysis of variance (ANOVA) was used to compare more than two groups. Student-Newman-Keuls test for post-hoc analysis was used to further evaluate the significance between the groups. A *p*-value of less than 0.05 was considered to be statistically significant.

## 3. Results

### 3.1. Vascular ACE mRNA Expression and Protein Levels

**Figure 1:**
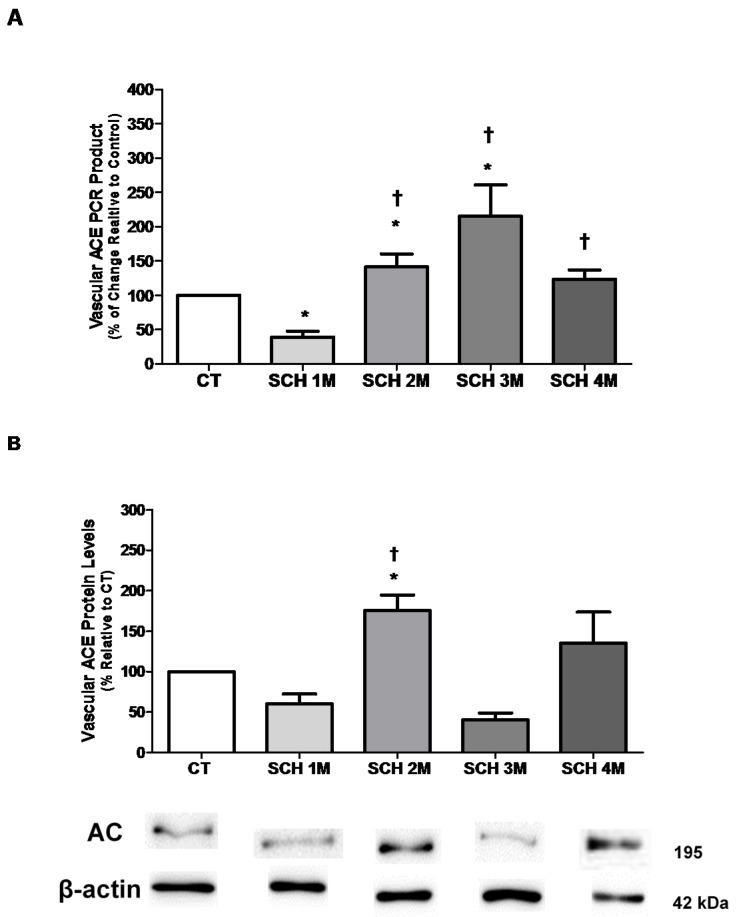
(**A**) Quantitative RT-PCR was used to analyze the aortic ACE mRNA expression profile from one to four months of age. Data represent values normalized against elongation factor 1-alpha and expressed as percentage (%) of change, relative to the CT. Error bars represent the standard error of the mean (SEM) for an average of six animals per group. ANOVA followed by Newmans-Keuls post-hoc test were used to determine the significant differences among groups; (**B**) ACE protein levels in aortic tissue from Syrian cardiomyopathic hamsters (SCH) between one month and four months of age. The levels of ACE protein were determined by Western blot from aortic homogenates. Data represent values normalized against β-actin and are expressed as percentage, relative to CT. The results represent the mean and standard error of the mean (SEM) of three animals per group. Analyses of variance (ANOVA), followed by the Newmans-Keuls post-hoc tests, were used to determine significant differences among the groups. * *p* < 0.05, when compared to age-matched CT. † *p* < 0.05, when compared to one-month-old SCH. The blots are representative bands from the indicated time points analyzed by Western blots.

Figure 1A shows vascular mRNA expression of ACE in SCH from one to four months of age. ACE mRNA expression was lower in one-month-old SCH than in age-matched CT hamsters (39% ± 9% in SCH vs. 100% in CT; *n* = 7, *p* < 0.05), but increased to 141% ± 19% at two months (*n* = 7, *p* < 0.05), and to 215% ± 45% at three months (*n* = 5, *p* < 0.05), before reaching a plateau of 123% ± 14% at four months. ACE protein levels in the vasculature of SCH from one to four months of age were evaluated by Western blot as shown in Figure 1B. ACE protein levels were higher in two-month-old (176% ± 19%, *p* < 0.05, *n* = 3) than in one-month-old SCH (60% ± 12%, *n* = 3, *p* < 0.05). No statistically significant differences were observed for ACE protein levels between SCH and CT hamsters at one, three or four months of age (Figure 1B).

### 3.2. Vascular eNOS mRNA Expression and Protein Levels

**Figure 2:**
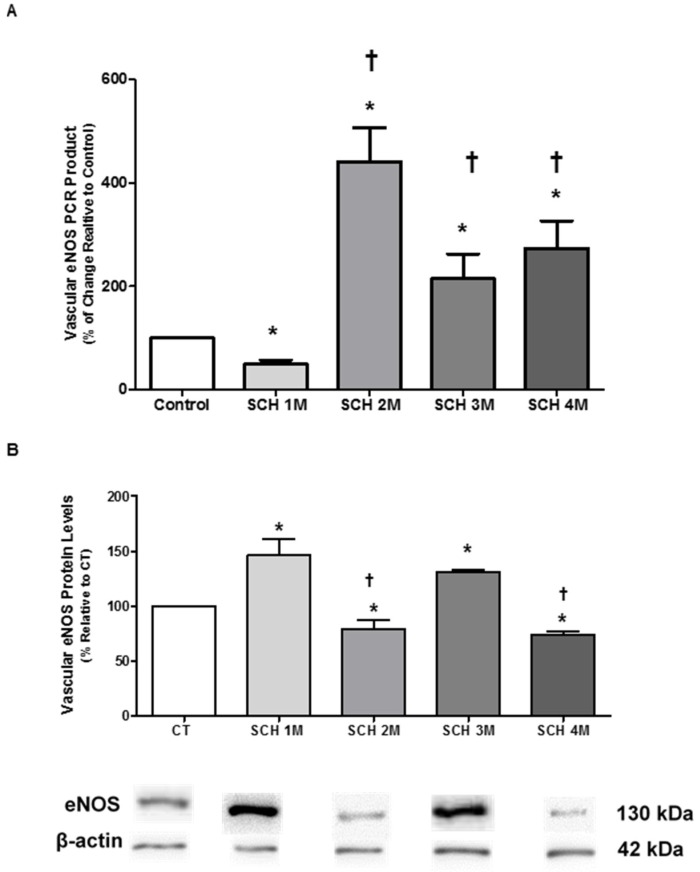
(**A**) The aortic eNOS gene expression profile from one- to four-month-old SCH hamsters was analyzed by quantitative RT-PCR. Data represent values normalized against elongation factor 1-alpha and expressed as percentage (%) of change, relative to the CT. Error bars show the standard error of the mean (SEM) for an average of seven animals per group. ANOVA followed by Newmans-Keuls post-hoc test were used to determine the significant differences among groups. * *p* < 0.05, when compared to CT; † *p* < 0.05, when compared to one-month-old SCH; (**B**) Aortic eNOS from Syrian cardiomyopathic hamsters (SCH) between one month and four months of age. Extracted proteins were analyzed by Western blot. Data represent values that were normalized against β-actin and expressed as percentage, relative to controls (CT). The results represent the mean and standard error of the mean (SEM) for three or four animals per group. Analyses of variance (ANOVA) followed by the Newmans-Keuls post-hoc tests were used to determine significant differences among the groups. * *p* < 0.05, when compared to age-matched CT. † *p* < 0.05, when compared to one-month-old SCH. The blots are representative bands from the indicated time points analyzed by Western blots.

**Figure 3:**
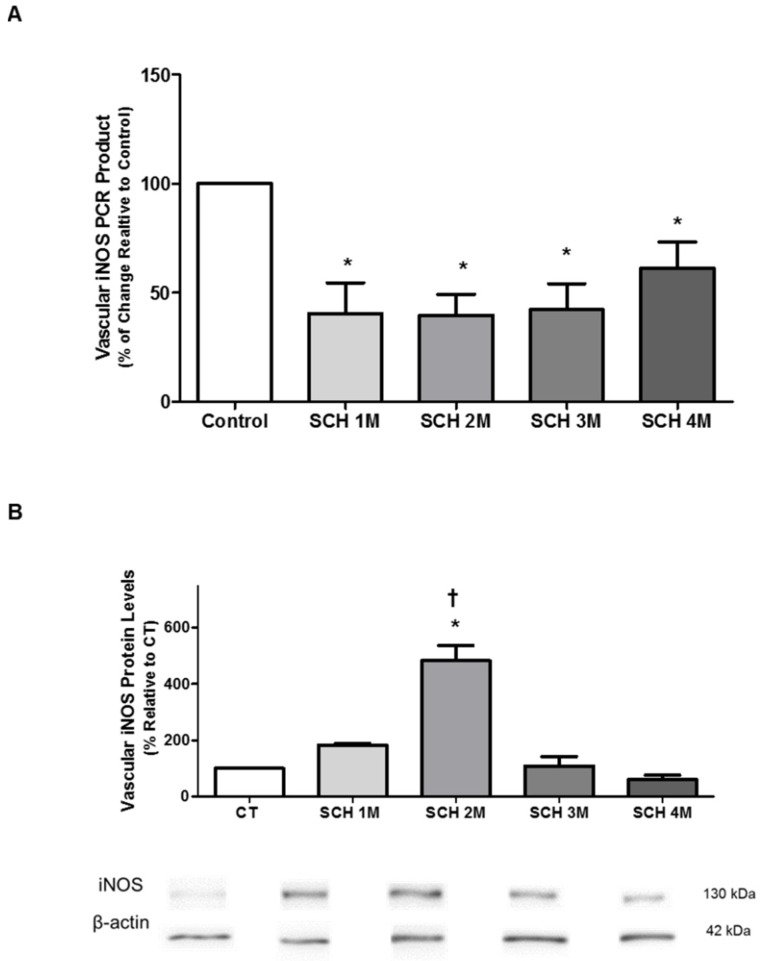
(**A**) Quantitative RT-PCR was used to analyze the aortic iNOS mRNA expression profile from one to four months of age. Data represent values normalized against elongation factor 1-alpha and expressed as percentage (%) of change, relative to CT. Error bars show the SEM for an average of five animals per group. ANOVA followed by Newmans-Keuls post-hoc test were used to determine the significant differences among groups. * *p* < 0.05, when compared to CT; (**B**) Vascular iNOS protein levels in aortic tissue from Syrian cardiomyopathic hamsters (SCH) between one month and four months of age. The levels of iNOS protein from aortic homogenates were determined by Western blot. Data represent values that were normalized against β-actin and expressed as percentage, relative to CT. The results represent the mean and standard error of the mean (SEM) for three to five animals per group. Analyses of variance (ANOVAs) followed by the Newmans-Keuls post-hoc tests were used to determine significant differences among the groups. * *p* < 0.05, when compared to age-matched CT. † *p* < 0.05 when compared to one-month-old SCH. The blots are representative bands from the indicated time points analyzed by Western blots.

In Figure 2 and Figure 3, we determined the mRNA expression and protein levels of eNOS and iNOS in the vasculature of young SCH to correlate these parameters with lower levels of nitric oxide previously reported by our group [9,11]. Figure 2A illustrates that eNOS mRNA expression was lower in one-month-old SCH than in age-matched CT hamsters (49% ± 8% in SCH vs. 100% in CT; *n* = 8, *p* < 0.05), but increased at two, three and four months of age, surpassing CT levels (442% ± 65% at two months; 214% ± 48% at three months; and 274% ± 53% at four months; *n* = 6–8, *p* < 0.05). Protein levels of eNOS between one and four months of age are presented in Figure 2B. These levels were higher in one-month-old and three-month-old SCH (146% ± 15% and 131%± 3%, respectively; *n* = 7, *p* < 0.05) than in age-matched CT hamsters (100%). In two- and four-month-old SCH, by contrast, eNOS protein levels were lower than in age-matched CT hamsters (87% ± 1% at two months and 73% ± 3% at four months vs. 100% for CT, *p* < 0.05).

### 3.3. Vascular iNOS mRNA Expression and Protein Levels

Figure 3A shows that mRNA expression of iNOS was reduced in SCH at all ages tested (40% ± 14% at one month, *n* = 7, *p* < 0.05; 40% ± 10% at two months, *n* = 13, *p* < 0.05; 42% ± 12% at three months, *n* = 5, *p* < 0.05; and 61% ± 12% at four months, *n* = 7, *p* < 0.05), when compared to age-matched CT hamsters (100%, *n* = 11, *p* < 0.05). Vascular iNOS protein levels, however, increased in SCH at two months of age (483% ± 55%, *n* = 5, *p* < 0.05) when compared to age-matched CT hamsters (100%, n = 3, p < 0.05) and when compared to SCH at one, three and four months of age (Figure 3B).

### 3.4. Effect of NO Bioavailability on Vascular ACE Activity

**Figure 4:**
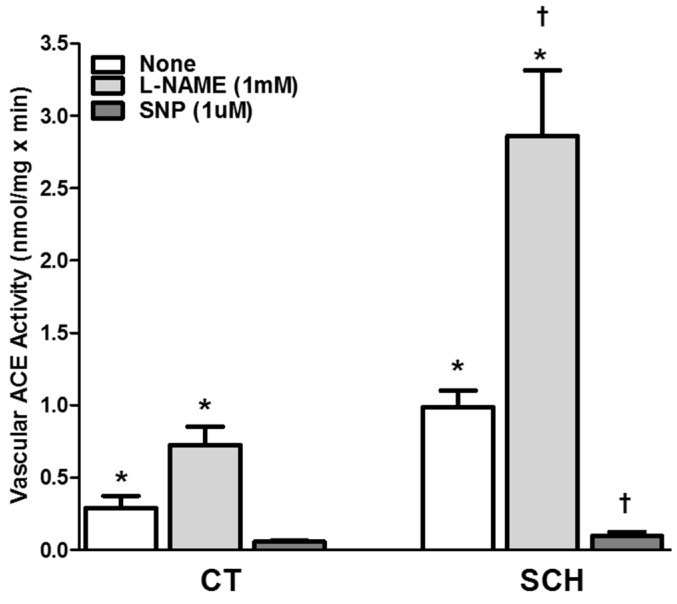
Effect of 6 h incubation with l-NAME (1 mM) and SNP (1 µM) on aortic ACE activity in two-month-old Syrian cardiomyopathic (SCH) and control (CT) hamsters. The results represent the mean and standard error of the mean (SEM) of seven to 22 animals per group. Analyses of variance (ANOVAs) followed by the Newmans-Keuls post-hoc tests were used to determine significant differences among the groups. * *p* < 0.05, when compared to age-matched CT. † *p* < 0.05, when compared to one-month-old SCH.

Evaluation of the effect of NO bioavailability on vascular ACE activity after a 6 h incubation period with l-NAME and SNP in aortic tissue from two-month-old SCH revealed that ACE activity was higher in SCH (0.99 ± 0.11 nmol/mg × min, *n* = 11, *p* < 0.05) than in age-matched CT hamsters (0.29 ± 0.09 nmol/mg × min, *n* = 22, *p* < 0.05; Figure 4). Incubation with l-NAME, a nitric oxide synthase inhibitor, increased ACE activity from 0.99 ± 0.11 to 2.86 ± 0.45 nmol/mg × min in SCH (*n* = 7, *p* < 0.05). The same pattern was present in age-matched CT hamsters, where ACE activity increased to 0.72 ± 0.13 nmol/mg × min after l-NAME incubation. By contrast, incubation of aortic homogenates with the NO releaser SNP reduced ACE activity in SCH to 0.10 ± 0.03 nmol/mg × min (*n* = 8, *p* < 0.05).

### 3.5. Effect of NO Bioavailability on Vascular ACE mRNA Expression

Similar effects on ACE mRNA expression were observed after SNP or l-NAME incubation of aortic homogenates from two-month-old SCH (Table 2). ACE mRNA expression was higher in SCH (141% ± 19%) than in age-matched CT hamsters (100%), and increased to 442% ± 103% (*n* = 6, *p* < 0.05) with l-NAME, while decreasing to 32% ± 9% (*n* = 6, *p* < 0.05) with SNP.

## 4. Discussion

The purpose of this study was to evaluate the developmental time course of ACE, eNOS, and iNOS in the vasculature of young SCH in order to correlate alterations in these variables with the onset of vascular modifications that precede cardiac deterioration in this animal model. Our combined results indicate that in the aorta of two-month-old SCH, ACE over-activation (increased levels of mRNA, protein, and activity) occurs concomitantly with a decrease in eNOS protein levels and an increase in iNOS protein levels. The combination of vascular hyper-reactivity due to RAS over-activation and reduced NO bioavailability resulting from NOS alterations may be crucial in the etiology of the vascular abnormalities present in young SCH. Moreover, by modulating ACE protein expression, activity, or both, oxidative stress may underlie the RAS over-activation present in SCH at two months of age, before the appearance of HF [9,11,12].

Our research group has proposed that the vasculature plays a prominent role in the etiology of HF in SCH because vascular alterations precede the clinical manifestations of the condition [9,11,12]. This contention is confirmed by the finding that at two months of age, vascular ACE mRNA expression, protein levels, and ACE activity increase simultaneously. ACE overexpression may enhance vascular reactivity and eventually trigger cardiac deterioration. Moreover, vascular ACE overexpression may underlie the endothelial dysfunction found in these animals at two months of age [9,19]. Indeed, chronic treatment of young SCH (from one to six months of age) with a combination of enalapril (angiotensin-converting enzyme inhibitor, ACEI) and losartan (angiotensin receptor blocker, ARB) improves vascular function and retards the development and progression of dilated cardiomyopathy, suggesting that vascular RAS plays a role in the onset of HF [20]. Similarly, in the mRen2.Lewis rat model of tissue renin overexpression, a dual blockade of RAS with an ACEI and an ARB in young pre-hypertensive rats prevents the development of hypertension and the appearance of diastolic dysfunction [21]. In patients with coronary artery disease, overexpression of tissue ACE disrupts the angiotensin II/bradykinin balance, resulting in endothelial dysfunction [22]. Overexpression of tissue ACE also has been directly related to the progression and disruption of atherosclerotic plaque and to endothelial damage in patients with coronary artery disease [23]. Thus, increased expression, protein levels, and activity of ACE may be a trigger for vascular deterioration, and a major factor in the etiology of cardiac damage.

The finding of reduced ACE expression in SCH at one month was unexpected, considering that ACE protein levels are similar in SCH and CT at this stage. Knowing that the SCH exhibits a deletion of the δ-sarcoglycan gene [24], however, the contribution of genetic alterations to the etiology of cardiac and vascular abnormalities should also be considered. It is possible that the deletion of the δ-sarcoglycan gene interferes with or delays, at the molecular level, the expression and development of all the components of the vascular RAS, including the expression of ACE at this early stage.

At two months of age, up-regulation of vascular eNOS mRNA may appear as a compensatory mechanism to maintain homeostasis because endothelial dysfunction is present at this early stage. This up-regulation, however, is not followed by increased protein levels. By contrast, reduced protein levels of eNOS are found concurrently with increased vascular iNOS protein levels, suggesting that the NOS system is ineffective in increasing NO levels for maintaining homeostasis when RAS is over-activated. The reduction of eNOS protein levels at two months, despite increased eNOS mRNA levels, confirms that regulation of this protein expression is not at the transcriptional (mRNA synthesis) or post-transcriptional (mRNA stability) levels because the amount of mRNA does not change at the same time as the protein level. Rather, the eNOS protein level at two months is regulated at the translational (decrease in protein synthesis) or post-translational (increase in protein degradation) levels. This pattern of regulation of protein expression, which does not correlate with mRNA expression, has been reported previously [25,26,27,28]. Whereas many proteins have a half-life ranging from minutes to days, mRNAs may degrade within hours (2–7 h), confirming that a correlation will not always be observed between mRNA and protein levels [29]. Mechanisms related to ubiquitin ligases, proteases, phosphorylation levels, and even cellular events, such as endocytosis and autophagy, may control protein half-lives, which differ from other mechanisms, such as microRNAs that regulate mRNA levels. Comini and colleagues [30] reported reduced eNOS protein levels in the aortas of rats with overt HF, but unlike in their adult rodent model, cardiac function is still intact in two-month-old SCH, suggesting that vascular eNOS alterations are involved in the etiology of HF and precede cardiac deterioration. The mechanisms underlying elevated iNOS protein levels at two months only are still unknown, and are beyond the scope of the current study. The selective increase in iNOS protein levels correlates, however, with increased oxidative stress and vascular abnormalities found in SCH at this stage [11,12]. Similarly, increased protein levels of iNOS also have been found in the vasculature of Sprague Dawley rats, as a result of chemically-induced oxidative stress [31].

That ACE protein expression and activity are attenuated by the NO donor SNP and enhanced by NOS suppression with l-NAME support the hypothesis that NO bioavailability modulates vascular ACE in SCH. Reduced NO bioavailability due to ROS overproduction secondary to NOS alterations may be responsible for the transient increase of ACE activity at two months of age, suggesting that oxidative stress is likely to underlie RAS over-activation at this stage. Similar to our findings for the aorta, we found in a previous study of the coronary circulation in young SCH that bradykinin-dependent relaxation occurs through an l-NAME-sensitive pathway, and that pre-treatment with the Ang II type 1 receptor blocker losartan or the antioxidant *N*-acetylcysteine improves bradykinin-induced relaxation [13]. These results also suggest that vascular RAS and oxidative stress underlie endothelial dysfunction and vascular over-reactivity in the early stages of SCH cardiomyopathy. Oxidative stress has been related to up-regulation of ACE activity in the aorta of Wistar-Kyoto rats after long-term inhibition of NO synthesis with l-NAME [32]. Quenching of NO by reactive oxygen species leads to activation of the vascular wall and has been implicated in the occurrence of inflammation, hypertension, atherosclerosis, and restenosis following vascular injury [33,34].

A limitation of this study is that the SCH is a genetic model of cardiomyopathy that exhibits a deletion of the δ-sarcoglycan gene. This genetic alteration causes a disruption in the sarcomere-sarcolemma complex, and in the signaling pathway downstream to the complex. When analyzing experimental data derived from this animal model, additional potential effects that the genetic modifications may have on the heart and vasculature must be considered. Changes in the sarcomere-sarcolemma complex may decrease contractility in both cardiac and vascular smooth muscle cells [35]. In young null mice, deletion of the δ-sarcoglycan gene has been linked to vascular remodeling without alterations in cardiac function [24]. In older hamsters, deletion of the δ-sarcoglycan gene results in cardiac alterations that are sensitive to treatment with α-blockers and calcium channel blockers [36,37]. Thus, a limitation of this study is that the SCH is a genetic model of cardiomyopathy that exhibits a deletion of the δ-sarcoglycan gene. This genetic alteration causes a disruption in the sarcomere-sarcolemma complex, and in the signaling pathway downstream to the complex. When analyzing experimental data derived from this animal model, additional potential effects that the genetic modifications may have on the heart and vasculature must be considered. Changes in the sarcomere-sarcolemma complex may decrease contractility in both cardiac and vascular smooth muscle cells [35]. In young null mice, deletion of the δ-sarcoglycan gene has been linked to vascular remodeling without alterations in cardiac function [24]. In older hamsters, deletion of the δ-sarcoglycan gene results in cardiac alterations that are sensitive to treatment with α-blockers and calcium channel blockers [37]. Thus, although not evaluated in the current study, the contribution of genetic alterations to the etiology of cardiac and vascular abnormalities should be also considered.

## 5. Conclusions

The present work provides evidence that the increased vascular reactivity present in two-month-old SCH is secondary to the over-activation of RAS that results from reduced NO bioavailability. The combination of lower eNOS protein levels and higher iNOS protein levels may contribute to the known reduction of NO bioavailability, and to the appearance of endothelial dysfunction documented previously by our group [9,11,12]. Moreover, reductions in NO at the vascular level in young SCH may be central to triggering transient vascular ACE activation and to perpetuating endothelial dysfunction by promoting oxidative stress. Together, these factors are likely to play a significant role in the development of endothelial dysfunction and vascular hyper-reactivity in SCH during the early stages of HF, and to eventually trigger cardiac deterioration in this animal model of dilated cardiomyopathy. The results of this study may contribute to the design of more effective therapies for simultaneously targeting RAS and oxidative stress in HF at the earliest stages of the disease.

## Figures and Tables

**Table 1:** Primers design and characteristics.

Primer	RNA Source	Sequence	Accession Number	Tm (°C)	Product Size (bp)
ACE sense	Hamster	5′-CCACAGTCTCAACCTGCTCA-3′	AB212958	55	181
ACE anti-sense	Hamster	5′-GCTCCACCACTCTTGGTTGT-3′			
eNOS sense	Hamster	5′-GGTCAACTATTCCCTGTCC-3′	AJ863053	57.5	287
eNOS anti-sense	Hamster	5′-ACACCACATCATACTCATCC-3′			
iNOS sense	Hamster	5′-ATATACCTCCTGAGTGAAG-3′	DQ355357	55	137
iNOS anti-sense	Hamster	5′-GGTCCTTGGTTGTAGATA-3′			
EF1-α sense	Rat	5′-ATTGTTGCTGCTGGTGTTG-3′	NM_175838	55	161
EF1-α anti-sense	Rat	5′-TCGTATCTCTTCTGACTGTATGG-3′			

**Table 2:** Effect of 6 h incubation with l-NAME (1 mM) and SNP (1 µM) on vascular ACE mRNA expression in two-month-old SCH.

	Control	SCH	SCH + LNAME	SCH + SNP
ACE mRNA	100%	141% ± 19% *	442% ± 103% *^,^^†^	32% ± 9% *^,^^†^

The results represent the mean ± SEM of six to 13 animals per group. Analysis of variance (ANOVA), followed by Newmans-Keuls post-hoc tests, was used to determine significance among the samples studied. (*): *p* < 0.05 compared to age-matched controls (CT); (†): *p* < 0.05 compared to one-month-old SCH.

## Abbreviations

ACE	Angiotensin converting enzyme
ACEI	Angiotensin converting enzyme inhibitor
ARB	Angiotensin receptor blocker
cDNA	Complementary deoxyribonucleic acid
CT	Control hamster
DTT	dl-Dithiothreiol
eNOS	Endothelial nitric oxide synthase
HF	Heart failure
iNOS	Inducible nitric oxide synthase
NADPH	Nicotinamide adenine dinucleotide phosphate (reduced form)
NO	Nitric Oxide
PMSF	Phenylmethanesulfonyl fluoride
RAS	Renin angiotensin system
RNA	Ribonucleic acid
RT-PCR	Reverse Transcriptase-Polymerase chain reaction
SCH	Syrian Cardiomyopathic hamster
